# Zn^2+^ as a secondary messenger for exogenous redox potential sensed through Chemosensory Zinc-Binding (CZB) protein domains

**DOI:** 10.64898/2026.07.03.735341

**Published:** 2026-07-07

**Authors:** Kailie Franco, Kendal G. Cooper, Clara Hyunyoung Shin, Olivia Steele-Mortimer, Arden Baylink

**Affiliations:** 1 Washington State University, Department of Veterinary Microbiology and Pathology, Pullman, WA 99164; 2 Laboratory of Bacteriology, Rocky Mountain Laboratory, National Institutes of Allergy and Infectious Diseases, National Institutes of Health, Hamilton, MT, 59840, USA.

## Abstract

Redox environments in nature are shaped by reactive oxygen species (ROS), oxygen availability, and metal ion chemistry, and exert profound effects on cell physiology and survival. While extensive work has characterized how cells resist oxidative damage, the mechanisms by which cells sense and navigate environmental redox conditions remain less well understood. Here, we identify a previously unrecognized and widespread mechanism of redox sensing in *Salmonella enterica* serovar Typhimurium mediated by the chemosensory zinc-binding (CZB) domain–containing receptor McpA. Using quantitative chemotaxis assays and live-cell imaging, we show that *S.* Typhimurium exhibits robust, concentration-dependent chemotaxis toward the neutrophil-derived oxidants HOCl and hydroperoxides, with attraction occurring at low, physiologically relevant concentrations below those that cause bactericidal effects, and this response requires McpA and its conserved zinc-binding cysteine. Whereas other Cys–Zn thiolate systems function through direct oxidation mechanisms, we find that the unique 3His,1Cys binding motif of CZBs responds to redox-dependent changes in Zn^2+^ speciation, whereby oxidizing conditions shift soluble, bioavailable Zn^2+^ into insoluble zinc precipitates. In this way, CZBs utilize the bioavailable Zn^2+^ pool as a secondary messenger of exogenous redox potential, and correspondingly, cells exhibit chemoattraction toward Zn^2+^-depleted environments, including sources of ROS, but also toward oxygen-rich conditions that provide a metabolic growth advantage. The broad phylogenetic distribution of CZB domains is consistent with this Zn^2+^-responsive mechanism being an ancient redox-sensing strategy, likely established early in bacterial evolution under changing planetary redox conditions and retained across diverse bacterial lineages.

## Introduction

Approximately 2.4 billion years ago, life on primordial earth was subjected to a mass extinction event driven by the appearance of high levels of molecular oxygen (O_2_), known as the Great Oxygenation Event (GOE) (1, 2). The increase in atmospheric O_2_ provided opportunities for more efficient metabolism through the use of O_2_ as a terminal electron acceptor, but also new and deadly sources of oxidative stress such as reactive oxygen species (ROS) (3). These chemicals, such as the superoxide anion (O_2_^.−^), hydroperoxides (ROOH), and hypochlorous acid (HOCl) can kill cells through lipid peroxidation, DNA damage, base oxidation and deamination, and protein carbonylation (4–6). All cells today bear the scars of this evolutionary bottleneck in the form of a sophisticated network of antioxidant enzymes and small-molecule reductants that strictly maintain intracellular redox homeostasis (7, 8). Further, cells engage in redox warfare through the deliberate production of ROS, as exemplified by neutrophils, which generate high concentrations of ROS through the respiratory burst to kill microbial pathogens (3–6). While an extensive body of research has elucidated a litany of strategies by which cells endure and combat oxidative stress, the sensory mechanisms that enable cells to perceive and respond to environmental redox conditions remain comparatively understudied (7, 9, 10).

Chemosensory zinc-binding (CZB) protein domains are found in all major bacterial lineages, suggesting that they originated before the diversification of extant bacterial phyla (11). As their name suggests, CZB domains bind a single Zn^2+^ ion with femtomolar-range affinity, ligated by a unique 3His, 1Cys motif not seen in any other system, yet the biological purpose and significance of their metallosensing function has remained enigmatic (11–17). Typically found as components of chemoreceptors and diguanylate cyclases, CZB domains serve as regulators of chemotaxis and c-di-GMP signaling, thereby controlling behaviors central to motility and navigation, lifestyle transitions, and colonization (11, 17). Prior studies have demonstrated that CZB proteins mediate behavioral responses to ROS, but seemingly contradictory results and perplexing impacts on behavior have made the biological function difficult to resolve (11–13, 15, 18, 19). Strikingly, the best-characterized CZB protein to date, the chemoreceptor transducer-like protein D (TlpD) of *Helicobacter pylori*, confers strong attraction to HOCl *in vitro*, paradoxically navigating the bacteria toward a potent bactericide (12, 20). Whether this surprising behavior is artifactual and/or a species-specific phenomenon is unknown. Further, some studies have proposed TlpD mediates repulsion from H_2_O_2_, another inflammatory ROS (15, 18). Together, these findings have left the conserved sensory function of CZB domains unresolved, raising the fundamental question of how Zn^2+^-binding regulates environmental sensing and what biological function underlies the widespread conservation of this metallosensory mechanism.

A current mechanistic model of CZB sensory function proposes that the Cys-Zn moiety is sensitive to direct oxidation by neutrophilic HOCl to form cysteine-sulfenic acid (Cys-SOH), resulting in detachment of the Cys from the zinc-binding core and a local unfolding of the domain that promotes release of the Zn^2+^ ion and signal transduction (11, 12). Interestingly, at the protein level, the conserved zinc-binding Cys exhibits chemoselectivity and is preferentially oxidized by HOCl and inefficiently oxidized by hydroperoxides (11–13). This mechanism of direct cysteine oxidation proposed for CZBs is analogous to that seen for zinc-finger proteins and other systems shown to undergo redox-dependent zinc release (11–13, 21), but some lines of evidence argue against this model being a universally conserved functions of CZBs. First, CZBs predate animal hosts, and are possessed by diverse bacteria, including environmental species not associated with animal hosts that would rarely encounter concentrated hypohalous acids like those that occur at sites of inflammation (11). Second, we recently showed that while the zinc-binding Cys mediates bacterial fitness *in vivo* during colitis, a disease state where the enteric environment has increased oxygenation as well as prevalent HOCl and other host-derived ROS, infection in a mouse model deficient in HOCl generation did not impact CZB-mediated colonization (22–25).

A major obstacle to defining the function and sensory mechanism of CZB domains is the lack of a clear understanding of the behavioral outputs they direct. Most studies have been conducted in *H. pylori*, which poses challenges for dissecting chemotaxis mechanisms due to its variable and fickle motility, limited genetic tractability, and requirement for growth in rich liquid media. These challenges limit the ability to perform chemotaxis assays in a defined chemical background and to clearly identify the effector species being sensed (12, 15, 19, 26, 27). In fact, much of the evidence for ROS chemorepulsion is based on analysis of swimming behavior, in which bacteria are immersed in a homogeneous bath of effector, rather than direct visualization that demonstrates the bacteria to be attracted or repelled by a defined chemical point source (15, 19). Moreover, redox-active effectors are inherently difficult to study because they are transient and experiments require careful control of the atmospheric background, which is technically challenging and not typically undertaken.

Herein, we employed a more genetically tractable and experimentally accessible model system that enables live-imaging of behavioral responses to test different models of CZB sensory mechanisms. We investigated the CZB-regulated chemoreceptor methyl-accepting chemotaxis protein A (McpA) from *Salmonella* Typhimurium, an orthologue of TlpD with similar domain architecture and approximately 20% sequence identity (12, 17, 22). We constructed a novel chemotaxis platform that enables for the first time direct visualization of bacterial redox taxis in response to injected effectors under strictly controlled atmospheric conditions, and, orthogonally, also a high-throughput swimming assay with automated tracking and analysis of hundreds to thousands of individual bacteria. Using these complementary approaches, we uncover a novel mechanism of redox-sensing mediated through depletion of soluble Zn^2+^ that we propose to be the conserved and ancestral function of CZB chemosensors.

## Results

### Salmonella Typhimurium exhibits concentration-dependent taxis to HOCl

**Fig. 1.**
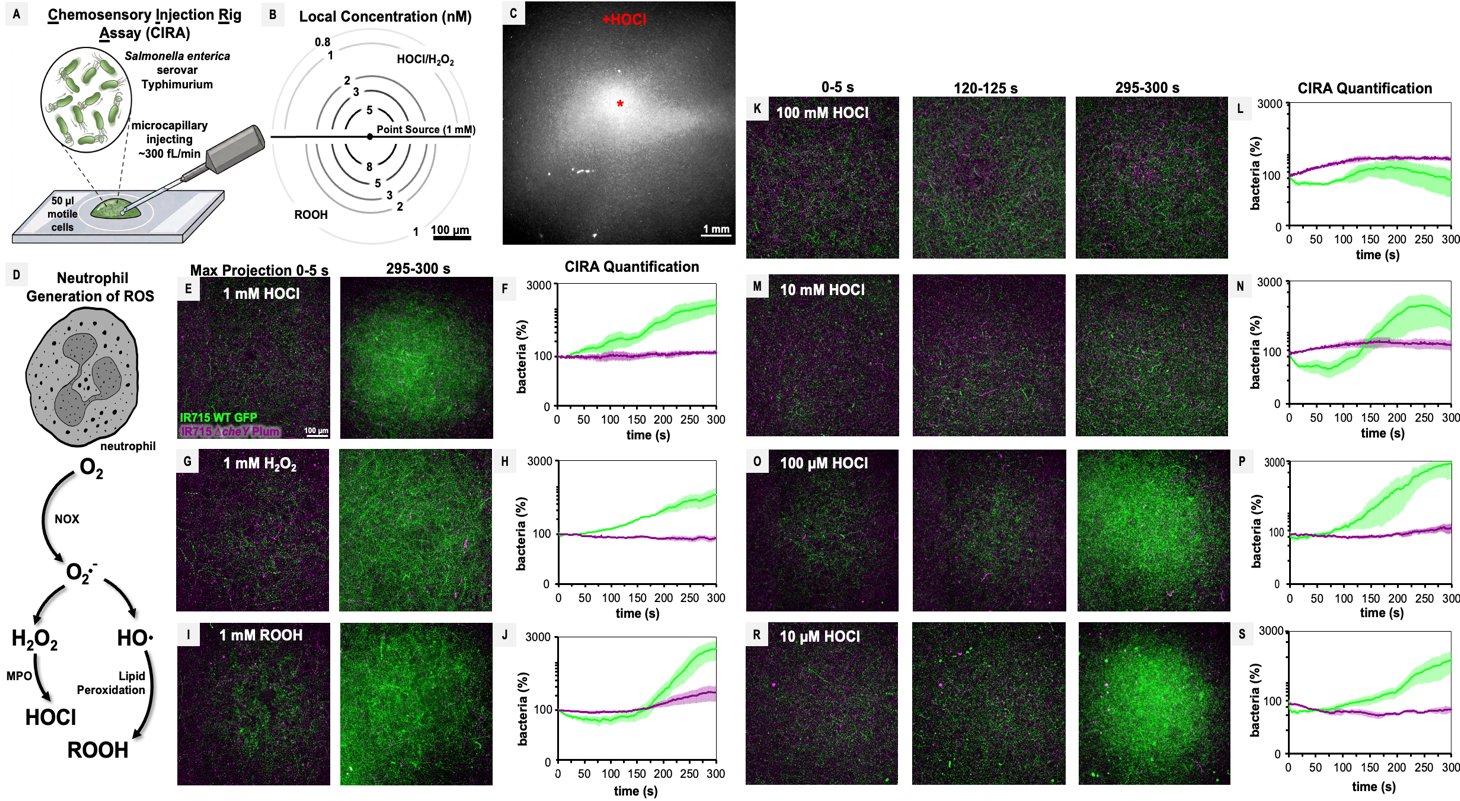
Neutrophil-derived reactive oxygen species drive chemoattraction in S. Typhimurium. A. Experimental design of CIRA. B. Diffusion modeling showing calculated local concentrations in CIRA experiments with ROS in relation to distance from the treatment source after 300 s of injection. C. Widefield view of S. Typhimurium attracted to a 1 mM HOCl injected treatment using CIRA at time 300 s. D. Overview of reactive oxygen species (ROS) production by neutrophils during the oxidative burst. E-J. Dual-channel imaging max projections of chemotactic responses to 1 mM ROS sources by WT *Salmonella* Typhimurium IR715 (green) and an isogenic Δc*heY* mutant (purple), at beginning and following 300 s of injection treatment, as indicated; shown right is the quantification of the number of cells in the field of view (FOV). K-R. Dual-channel imaging max projections of chemotactic responses to a titration of HOCl by WT *Salmonella* Typhimurium IR715 (green) and an isogenic Δc*heY* mutant (purple), as indicated. Graphs depict means with error calculated as SEM (n=4–5).

During infection, *Salmonella* Typhimurium induces colitis and leverages metabolic advantages to thrive in the inflamed gut, an environment where infiltrating neutrophils generate large quantities of ROS and transform the redox environment (23, 24, 28–30). Through NADPH oxidase, neutrophils generate H_2_O_2_ that is subsequently converted to the strong oxidant HOCl by myeloperoxidase; activated neutrophils can generate HOCl at rates approaching 134 mM min^−1^, and local HOCl concentrations within inflamed tissues or phagosomes have been estimated to reach up to 5 mM (Figure 1D). The oxidative burst also generates organic hydroperoxides (ROOH) via lipid peroxidation (Figure 1D), highly reactive oxidants capable of inducing cell lysis (32, 33). Hence, this bacterium regularly experiences high concentrations of these bactericidal oxidants, each possessing different reactivities, half-lives, and preferred oxidation targets, and no prior studies have tested if McpA mediates taxis to these oxidants.

To address this, we employed the Chemosensory Injection Rig Assay (CIRA, Figure 1A), which uses a glass microcapillary to continuously inject femtoliter volumes of effectors into a suspension of motile bacteria and monitor taxis responses through video microscopy (34, 35). In these experiments, swimming bacteria initially have a homogenous distribution, and exposure to treatment results in either no discernible change (null response), an influx of bacteria toward the treatment source (chemoattraction), or decrease in bacteria (chemorepulsion) (Figure S1A). Modeling of ROS diffusion in this assay predicts a steep, orders-of-magnitude concentration gradient from the source, such that even a 1 mM injection exposes most bacteria to physiologically relevant pico- to nanomolar ROS concentrations (Figure 1B).

We employed CIRA to model the neutrophil respiratory burst and study taxis responses to discrete ROS. We performed competition assays with fluorescently-tagged WT *Salmonella* Typhimurium IR715 (IR715 WT) and a Δ*cheY* knockout mutant, which is motile but blind to chemoeffector stimuli, and viewed their taxis responses simultaneously (Figure S1B). For a well-characterized attractant such as L-Ser, WT exhibits robust chemoattraction whereas Δ*cheY* remains randomly distributed (Figure S1B). CIRA experiments using 1 mM sources of HOCl, H_2_O_2_, and cumene hydroperoxide, which we used as a model organic hydroperoxide, all elicited chemoattraction, with more WT cells coming into the field of view over time, albeit a more diffuse response than seen for L-Ser (Figure 1C, E–J, Video S1). We also found *S.* Typhimurium strain SARA1 to be attracted to all three oxidants. (Figure S1C–E). Importantly, the distribution of the Δ*cheY* cells are not impacted by ROS treatments, and so the behavior cannot be due to a trapping mechanism or impairment of motility leading to aggregation of cells near the ROS source. These findings revealed that, like seen previously for *H. pylori*, *S.* Typhimurium is attracted to HOCl and also uncovered a previously unrecognized attraction to hydroperoxides.

While it is surprising the bacteria are attracted to sources of bactericidal oxidants, the consistency of this response across all three neutrophilic ROS led us to ask what concentrations elicit taxis. We performed CIRA experiments across a wide range of physiologically relevant HOCl concentrations and found responses to be concentration-dependent and adaptive (Figure 1K–R). At a source concentration of 100 mM, WT cells are initially repelled and then adapt after about four minutes and return to a random distribution (Figure 1K–L). At 10 mM this response is similar with a shorter adaptive period, with WT cells exhibiting chemorepulsion until approximately 2.5 minutes, and, thereafter, chemoattraction (Figure 1M–N). HOCl sources of 0.01–1 mM all promoted clear chemoattraction (Figure 1O–R). Hence, *S.* Typhimurium mediates differential taxis based on the concentration of exogenous ROS, with chemoattraction occurring only to levels in the nanomolar to picomolar range.

### McpA and other chemoreceptors regulate swimming behavior in response to HOCl

**Fig. 2.**
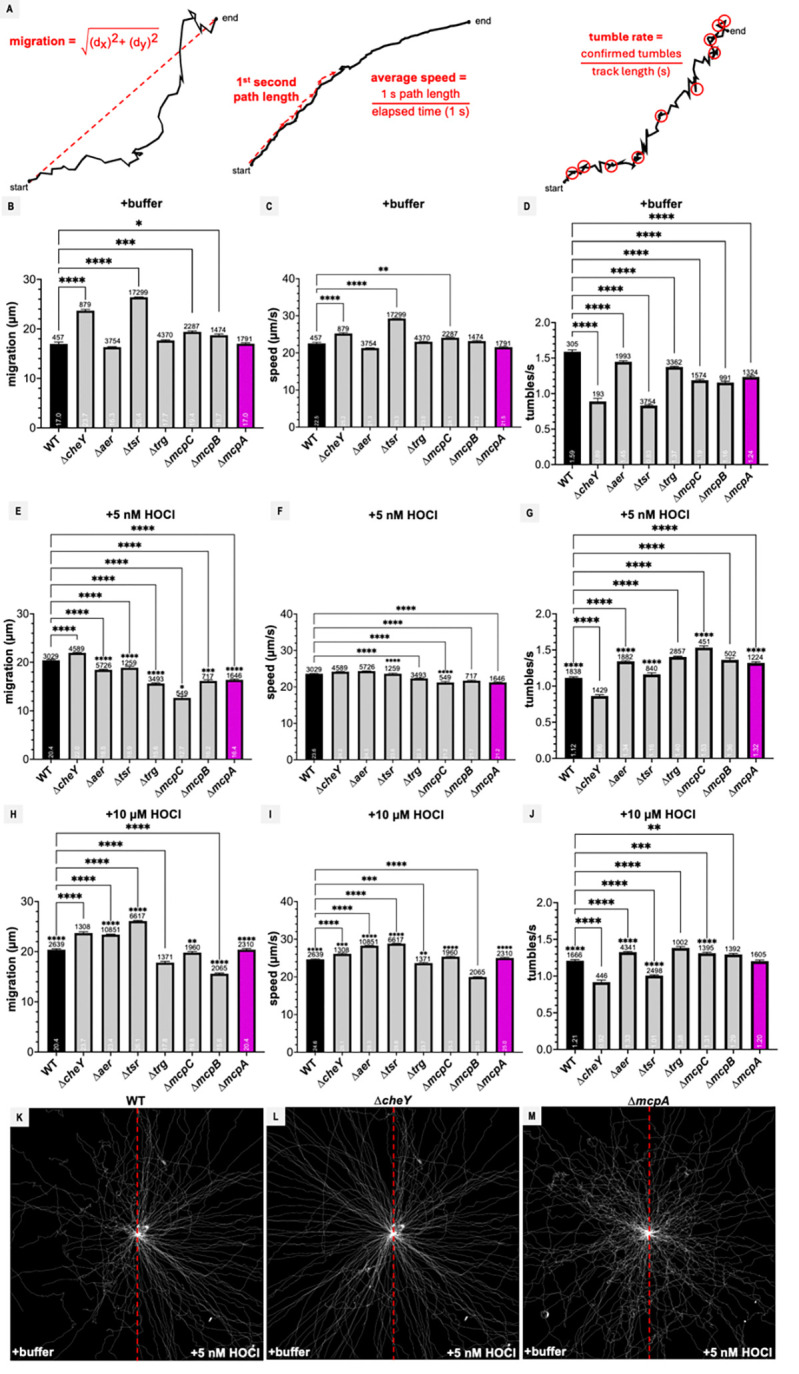
Bacterial swimming is responsive to neutrophil ROS. A. Representative single-cell bacterial tracks showing the methods of calculating migration, average first second speed and tumbling rate for bacteria swimming. B-D. Quantification of bacterial migration and speed over the first second of each track, as well as tumbles per second based on bathing cells in chemotaxis buffer (CB) and imaging swimming for 30 s, and quantification by automated single-cell bacterial tracking (see Methods). Numbers above bars denote number of tracks, while numbers within the bar are population means. E-J. Quantification of bacterial migration, speed and tumbles per second following bathing cells in 5 nM or 10 μM HOCl diluted in chemotaxis buffer. Graphs depict means with error bars as SEM. Statistical significances between strains, denoted by bars within each treatment group, were calculated using a Kruskal-Wallis test followed by Dunn’s multiple comparison tests, compared to the WT strain within that treatment group. Statistical significances for strain differences compared to buffer treatment version of the same strain are denoted by stars above the number of tracks and were calculated by unpaired t-tests (not significant, not noted; * p < 0.05, ** p < 0.01, *** p < 0.001, **** p < 0.0001). K-M. Representative spider plots of 150 of WT, Δ*cheY* and Δ*mcpA* bacterial tracks with and without HOCl addition.

Having established that *S.* Typhimurium is attracted to ROS sources through chemotaxis, we next sought to identify chemoreceptors responsible for regulating swimming behavior in response to exogenous HOCl. In the canonical model of chemotaxis, swimming bacteria alternate between runs and tumbles, and chemoattractants elicit decreased tumbling rates, which is also reflected in increased migration via longer uninterrupted runs (20, 36–41). To assess these behaviors in response to HOCl, we employed an assay in which bacteria are bathed in a homogenous treatment and then videos of swimming bacteria are collected and processed through an automated tracking pipeline to plot hundreds-to-thousands of individual bacterial swimming trajectories and quantify migration, speed, and tumbling frequency (Figure 2A) (12, 42). We performed this assay with WT and single receptor mutants to identify receptors involved in mediating HOCl taxis.

In these experiments we see that WT in the presence of 5 nM and 10 μM HOCl, concentrations in the range we calculate to stimulate chemoattraction in CIRA, shows increased migration and decreased tumbling rate, consistent with chemoattraction (Figure 2B, D–E, G–H, J). Swimming speed was similar across treatments, indicating HOCl at these concentrations does not impair motility (Figure 2C, F, I). In fact, we found that many receptor mutants show some degree of defect in overall taxis and also in HOCl response. Δ*mcpA,* Δ*aer,* Δ*trg* lost HOCl-responsiveness, as migration and tumbling rate is similar across treatments, while Δ*tsr,* Δ*mcpB,* and Δ*mcpC* show elevated tumble rates, i.e. an inverted response (Figure 2B, D–E, G–H, J). These defects in HOCl taxis in these mutants could be either from loss of direct HOCl-sensing or perhaps deterioration of nanoarray function; Aer and Tsr have also previously been implicated in ROS taxis and aerotaxis (23, 43–45). Together, in terms of regulation of swimming behavior in response to HOCl exposure, we see an attraction phenotype at low concentrations that is consistent with the localization toward ROS sources in CIRA, and multiple receptors, including McpA, appear to be involved in coordinating this behavior. Visually, this can be confirmed when viewing the bacterial tracks aligned to a central origin before and after HOCl addition (Figure 2K–M). For WT cells, the tracks appear more similar to Δ*cheY* upon treatment with HOCl (Figure 2K), with less direction changes, while Δ*cheY* and Δ*mcpA* have similar tracks regardless of treatment (Figure 2L–M).

### Salmonella Typhimurium tolerates ROS concentrations that elicit chemoattraction

**Fig. 3.**
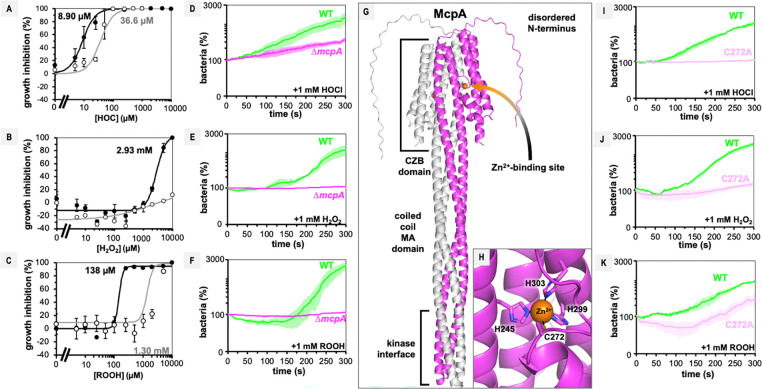
McpA mediates and chemotactic sensing of neutrophil ROS. A-C. *Salmonella* Typhimurium growth inhibition in the presence of increasing concentrations of ROS under normoxic (atmospheric, grey) or hypoxic (1% O_2_ + 10% CO_2,_ black) atmospheres at 37 °C; half maximal inhibitory concentration (IC_50_) is noted in grey and black for normoxic and hypoxic experiments, respectively (n=24). D-F. Quantification of dual-channel CIRA of responses to a 1 mM ROS point source by WT (green) versus Δ*mcpA* (magenta) as indicated. G. Alphafold3 model of the McpA homodimer, with one monomer colored in magenta. Zn^2+^ is modeled as an enlarged sphere in the CZB domain and colored orange. H. Close-up of the zinc-binding site within the CZB domain, with zinc-coordinating residues labeled. I-J. Quantification of dual-channel CIRA of responses to 1 mM ROS point source by WT (green) versus the Δ*mcpA* pmcpA C272A mutant (light pink), as indicated. Graphs depict means with error calculated as SEM (n=4–5).

The differential chemotactic responses across ROS concentration regimes led us to investigate how these ROS concentrations impact the growth of *S*. Typhimurium. Bacterial growth was assessed under both normoxic conditions, where aerobic respiration predominates, and hypoxia (1% O_2_ + 10% CO_2_), mimicking the low-oxygen environment of the gut (46, 47). As expected, higher O_2_ levels increase bacterial growth of this facultative anaerobe (Figure S2A–F). Of the neutrophil-derived ROS, HOCl is the most potent bactericide, with IC_50_ values of 8.9 μM and 36.6 μM under hypoxic and normoxic conditions, respectively (Figure 3A). Hydroperoxides are better tolerated with high micromolar to millimolar IC_50_ values (Figure 3B–C). Interestingly, under hypoxic conditions, low levels of H_2_O_2_ conferred a small growth benefit (Figure 3B). This observation is consistent with prior work showing *S.* Typhimurium can utilize H_2_O_2_ as an alternative electron acceptor under oxygen-limiting conditions (Figure 3B, Figure S2) (24, 25, 48, 49). Together, these data reveal that *S.* Typhimurium is attracted to low ROS levels it can tolerate, or even obtain a growth advantage from, and is repelled at high ROS concentrations that are inhibitory to growth.

### McpA and its zinc-binding Cys are required for ROS chemoattraction

As McpA was one of the receptors confirmed to be required for regulation of swimming behavior in response to HOCl, we next wanted to determine if this receptor is required for attraction to HOCl sources and other neutrophil-derived ROS. Using CIRA, we performed competition experiments with WT and Δ*mcpA* to 1 mM sources of HOCl and hydroperoxides, and found McpA to be necessary for full chemoattraction to HOCl, and hydroperoxide chemoattraction was completely dependent upon McpA (Figure 3D–F, Video S2). As a control, we also tested HOCl taxis for a genetic complement of McpA, and confirmed a similar phenotype as seen for WT (Figure S1F).

As chemoreceptors function as integrated signaling networks, and full gene deletions might exert pleiotropic effects, we aimed to dissect the mechanism of McpA-mediated ROS taxis at the single amino acid level. To address, as well as to probe whether the integrity of the zinc-binding core is required for ROS-sensing, we employed a C272A mutant, the cysteine residue universally conserved across all CZB proteins that coordinates the Zn^2+^ ligand and the target of direct HOCl oxidation in other systems (Figure 3G–H). In prior work, substitution of the conserved Cys was shown to reduce Zn^2+^-binding affinity by approximately an order of magnitude; however, because the binding affinity remains in the femtomolar range, the Zn^2+^ ligand is retained in the absence of a strong competing chelator (11, 14).

Using CIRA, we found that the C272A mutant lacks responsiveness to HOCl and shows diminished attraction to hydroperoxides (Figure 3I–K, Video S3). Interestingly, the C272A mutant actually shows a stronger defect in HOCl chemoattraction than Δ*mcpA*, but the reason behind this is unclear. Further studies of swimming behavior confirmed the C272A mutant to have defects in responsiveness to ROS in terms of migration and tumble rate, and some assays also showed phenotypic differences between the Δ*mcpA* and C272A mutant (Figure S2C–E, H–J, M–O). For example, H_2_O_2_ reduces tumbling rate for both WT and Δ*mcpA* but increases tumbling in the C272A mutant (Figure S2J). Hence, while the C272A mutant was found to have impaired ROS taxis, it was not a phenocopy of the Δ*mcpA*, which may be explained by the cysteine substitution weakening the Zn^2+^ affinity of the binding site rather than rendering it completely non-functional. Together, these data identify McpA as a mediator of ROS chemoattraction in *S.* Typhimurium and for the first time establish the conserved zinc-binding Cys to be integral for ROS taxis.

### Exogenous Zn^2+^ serves as a secondary messenger for local redox levels

The hypothesis that CZBs act as HOCl-sensors through preferential direct oxidation of the zinc-binding Cys thiolate predicts McpA would mediate taxis only to HOCl and not hydroperoxides that have been shown to be poorly reactive with the Cys-Zn moiety (13). That we observe similar responses to both HOCl and hydroperoxides dependent upon C272 argues there must be a different mechanism at play that mediates broad sensing of ROS. This led us to consider if the ROS-sensing function of McpA could be dependent upon binding of Zn^2+^.

**Fig. 4:**
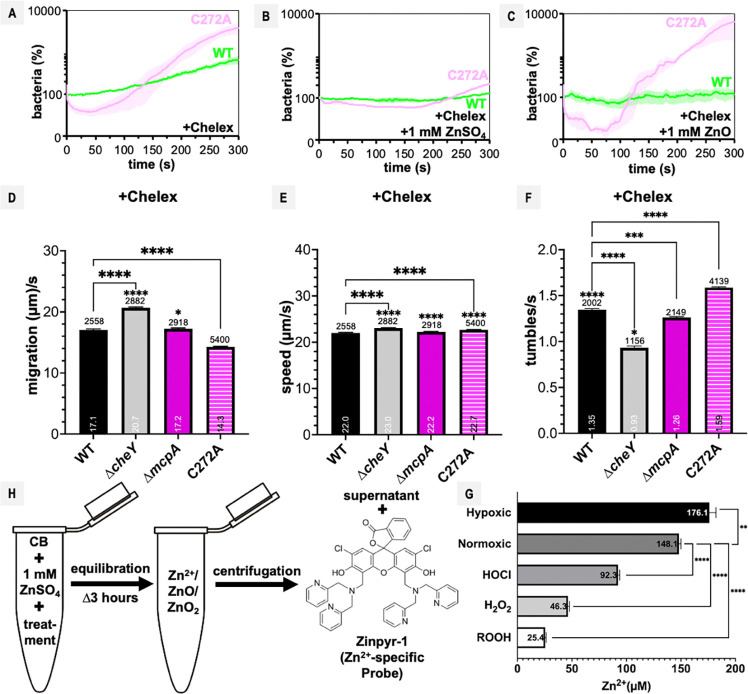
Soluble Zn^2+^ levels modulate McpA-mediated chemotaxis and bacterial swimming. A-C. Quantification of CIRA competition experiments between WT (green) and the C272A mutant (light pink) responding to solutions of different Zn^2+^ content, as indicated (n=3–5). D-F. Quantification of bacterial migration and speed over the first second of each track as well as tumbles per second based on bathing cells in chemotaxis buffer treated with Chelex for Zn^2+^ chelation and imaging swimming for 30 s, following by single-cell bacterial tracking. Numbers above bars denote number of tracks, while numbers within the bar represent numerical means. All graphs depict means with error calculated as SEM. Statistical significances between strains, denoted by bars within each treatment group, were calculated using a Kruskal-Wallis test followed by Dunn’s multiple comparison tests, compared to the WT strain within that treatment group. Statistical significances for strain differences compared to buffer treatment version of the same strain are denoted by stars above the number of tracks and were calculated by unpaired t-tests (not significant, not noted; * p < 0.05, ** p < 0.01, *** p < 0.001, **** p < 0.0001). G-H. Experimental overview for quantification of free Zn^2+^ in solution following treatment of ZnSO_4_ under various conditions, as indicated. Statistical significances were calculated by unpaired t-tests and are noted for each experiment in relation to normoxic CB.

To test this hypothesis, we first assessed whether McpA is responsive to changes in exogenous Zn^2+^ ion levels. Because Zn^2+^ is essential for cell viability, measurement of chemotaxis in fully Zn^2+^-depleted conditions are not possible because the cells rapidly lose motility (data not shown). However, we found that addition of tolerable, low micromolar levels of the Zn^2+^-chelator EDTA to the chemotaxis buffer nullifies attraction to HOCl, and sometimes results in repulsion close to the oxidant source (Figure S1G).To investigate how cells respond to gradients of Zn^2+^, we used CIRA and assessed taxis to Chelex-treated buffer, which substantially depletes zinc ions (50). In these experiments we saw the WT exhibits chemoattraction, and also that the C272A mutant exhibits an even greater attraction to zinc-depleted buffer (Figure 4A, Video S4). One explanation for this behavior is that the introduction of a local exogenous depletion in Zn^2+^ concentration drives the intracellular equilibrium to promote release Zn^2+^ by McpA, mediating an attraction response, and that due to the weaker Zn^2+^ binding affinity of the C272A mutant this effect is stronger in that background.

When Zn^2+^ was supplemented back into Chelex-treated buffer in the form of ZnSO_4_, WT and C272A were no longer responsive to the treatment (Figure 4B, Video S4). We also tested supplementation with ZnO and found in that case WT lost attraction, but C272A still demonstrated attraction to the buffer (Figure 4C, Video S4). One possible explanation is that ZnO generates a small pool of soluble Zn^2+^ sufficient to restore Zn^2+^ binding by the high-affinity WT receptor and abolish attraction, whereas these concentrations remain below the sensory threshold of the lower-affinity C272A mutant, allowing it to retain attraction to the buffer. In terms of swimming behavior, zinc depletion slightly lowers tumbling rate dependent upon Δ*mcpA* and C272A, consistent with an attraction response to low Zn^2+^ (Figure 4D–F). These findings together indicate that McpA is able to mediate responses to alterations in exogenous Zn^2+^, sans any ROS addition, and also that the receptor discriminates between Zn^2+^ and other zinc species.

Although Zn^2+^ itself is not redox active, environmental redox conditions influence zinc speciation by shifting the equilibrium between soluble, bioavailable Zn^2+^ and insoluble zinc-containing precipitates (51–55). Given the responsiveness of McpA to exogenous Zn^2+^ concentrations, we hypothesized that inert, Zn^2+^ could function as a secondary messenger reporting environmental redox potential, with oxidizing conditions depleting the extracellular soluble Zn^2+^ pool through precipitation to insoluble and zinc species that are either not recognized by McpA and/or are not bioavailable. To test if neutrophil-derived ROS impact Zn^2+^ ion availability in solution, we prepared aqueous solutions of 1 mM ZnSO_4_ and treated them with HOCl or hydroperoxides, and compared solutions prepared at normoxic and hypoxic conditions to test the influence of atmospheric O_2_ levels (Figure 4G). After three hours incubation, solutions were centrifuged to remove zinc precipitants and the remaining soluble Zn^2+^ ion content in solution was determined using the Zn^2+^-specific probe Zinpyr-1 (11, 56).

These experiments revealed that hypoxic solutions retain the highest amount of soluble Zn^2+^, followed by normoxic buffer, and ROS addition reduces the soluble, bioavailable Zn^2+^ pool even further by promoting formation of insoluble zinc species (Figure 4H). Although the precise composition of these precipitates was not determined, they are likely composed of zinc oxides, zinc hydroxide, and related zinc oxyhydroxide species (57, 58). Thus, there appears to be a reciprocal relationship with ROS addition and depletion of soluble Zn^2+^ ion (Figure 4K). The extent of Zn^2+^ depletion followed the order ROOH > H_2_O_2_ > HOCl > O_2_, suggesting that zinc precipitation is governed less by oxidizing potential alone and more by each oxidanťs ability to promote formation of insoluble Zn–O-containing aggregates (59, 60). Together, these data demonstrate that both ROS and O_2_ drive depletion of soluble Zn^2+^, consistent with an equilibrium process linking exogenous Zn^2+^ availability to the cellular Zn^2+^ pool, which is sensed by McpA to drive chemotaxis.

### McpA mediates attraction to O_2_ sources

After learning that McpA is responsive to exogenous Zn^2+^ ion levels in solution, and that this can be modulated not only by ROS, but also by O_2_ content, we wondered if McpA might mediate taxis in response to different levels of oxygenation, i.e. aerotaxis. Chemotaxis experiments are typically performed under normoxic (i.e., 21% O_2_) conditions, which obscures aerotaxis behaviors, as regulating oxygen content can be challenging or incompatible with certain microfluidics assays. To overcome this, we built a custom hypoxic chamber designed to house our CIRA setup, making it the first system, to our knowledge, where live aerotaxis responses can be viewed in real time in response to microinjection of a defined effector treatment source.

**Fig. 5:**
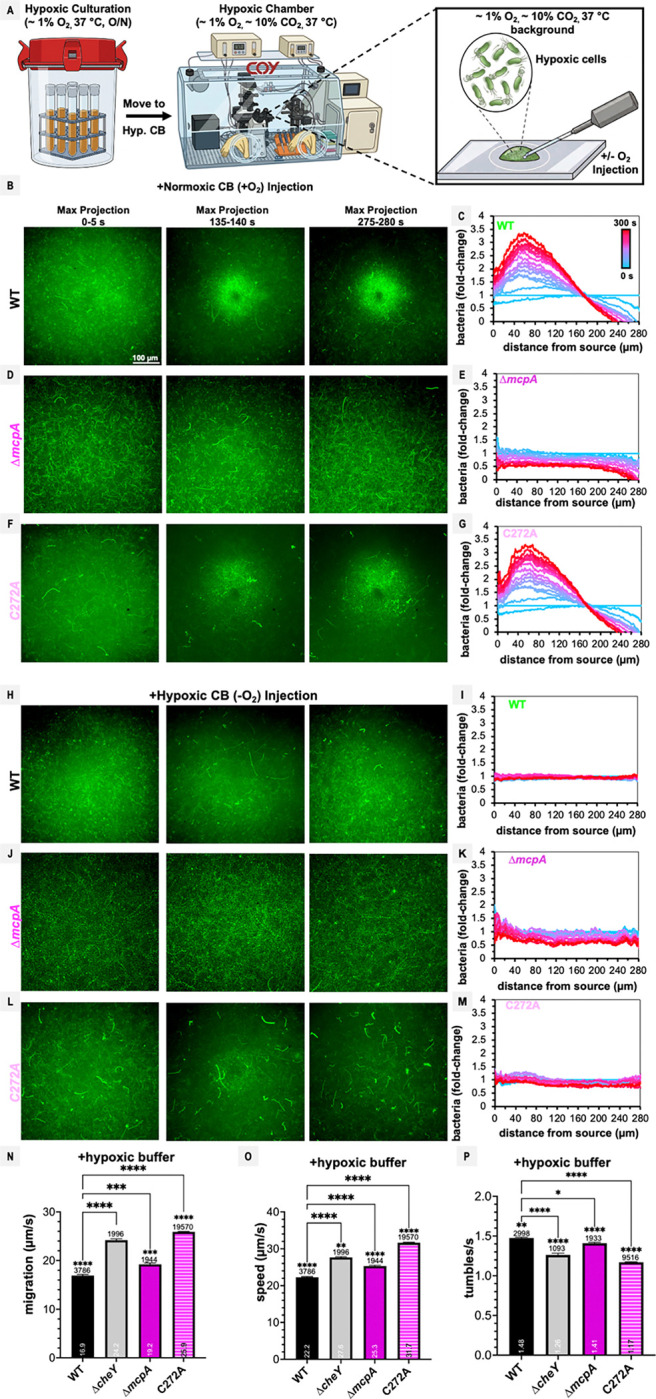
McpA mediates aerotaxis. A. Experimental design for CIRA under hypoxic conditions. B-G. Analysis and quantification of chemotactic responses under hypoxia to injected treatments with buffer equilibrated at normoxic conditions, as indicated. Shown are max projections at times 0–5, 135–140, and 275–280 s post-treatment, accompanied by measurements of bacterial population distribution over time. The initial uniform population distribution in these plots is indicated with a blue line (time 0), and the final mean distributions with the red line (time 280 s), with the mean distributions between these displayed as a blue-to-red spectrum at 20 s intervals. H-M. Analysis and quantification of chemotactic responses under hypoxia to hypoxic equilibrated buffer. N-P. Quantification of bacterial swimming behavior in hypoxic conditions. Graphs depict means with error calculated as SEM (n=4–5). Statistical significances between strains, denoted by bars within each treatment group, were calculated using a Kruskal-Wallis test followed by Dunn’s multiple comparison tests, compared to the WT strain within that treatment group. Statistical significances for strain differences compared to buffer treatment version of the same strain are denoted by stars above the number of tracks and were calculated by unpaired t-tests (not significant, not noted; * p < 0.05, ** p < 0.01, *** p < 0.001, **** p < 0.0001).

We used this unique setup to test the response of cells cultured under hypoxic conditions that mimic the native gut environment of *Salmonella* (1% O_2_ and 10% CO_2_) to an injected source of normoxic buffer (Figure 5A, Figure S3). Remarkably, S. Typhimurium exhibits strong attraction to elevated O_2_ that is entirely dependent upon McpA, with the C272A mutant retaining attraction similar in overall magnitude to the WT but with a cell population more diffuse (Figure 5B–G, Video S5). In contrast, cells exhibit no response to injected hypoxic buffer, supporting that the attraction is in response to the elevated O_2_, and presumably the locally lowered Zn^2+^ ion concentration (Figure 5H–M, Video S6). Swimming behavior is relatively consistent with the results from CIRA (Figure 5N–P). Under hypoxic conditions the WT shows lower migration and higher tumbling rate than under normoxic conditions indicative of a repulsion from lower O_2_ levels; Δ*mcpA* has slightly higher migration and lower tumbling rate than WT, supporting a role for McpA in mediating the swimming behavior, and C272A had migration and tumbling rate similar to Δ*cheY*, suggesting, potentially, that its lowered zinc-binding affinity is less able to distinguish the differential Zn^2+^ levels in hypoxic versus normoxic conditions, although tumbling rate is indeed lowered for this mutant under hypoxia (Figure 2, Figure 5M–O, Fig. S2). Together, these results confirm that McpA mediates aerotaxis toward elevated O_2_. As expected, normoxic conditions provide a substantial growth benefit for the bacterium, providing a biological rationale for this behavior (Figure 3).

**Fig. 6:**
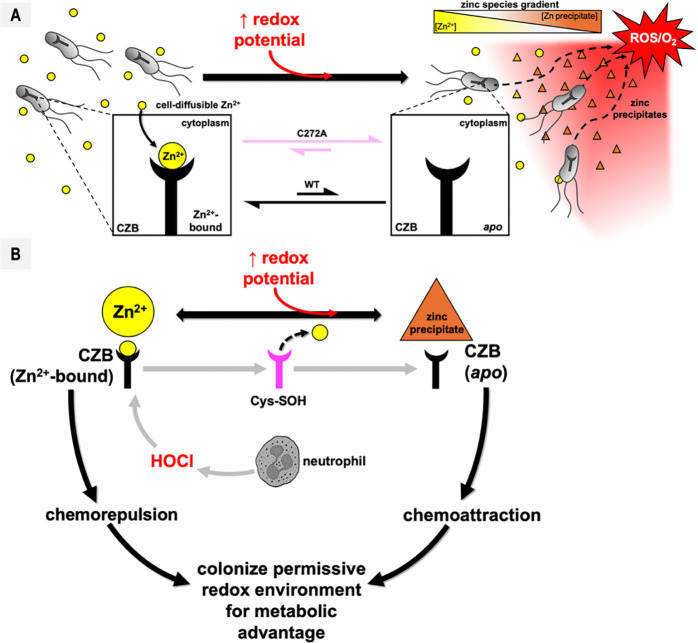
Model for CZB-mediated sensing of redox potential through exogenous Zn^2+^ as a secondary messenger. A. An environment with increased redox potential can cause Zn^2+^ (yellow circles), which can diffuse in and out of the bacterial cell, to form zinc precipitates (orange triangles) that can no longer enter the cell, thereby creating a Zn^2+^ gradient. When exogenous Zn^2+^ levels are depleted, this shifts the CZB domain toward an apo state (black equilibrium arrows). In the case of the C272A mutant, the CZB domain has weakened affinity (light pink equilibrium arrows) and thus is more readily able to release its Zn^2+^ ligand. In the case of the C272A mutant, CZB domain has approximately 10x less affinity for Zn^2+^ (light pink equilibrium arrows) and thus is more readily able to release its Zn^2+^. B. Release of the Zn^2+^ ligand promotes a chemoattraction response. Conversely, at lower redox potential and higher Zn^2+^ levels, the CZB population is mostly zinc-bound, promoting a chemorepulsion response. In host-associated CZB-containing bacteria, HOCl generated by neutrophils can mediate direct cysteine oxidation (grey arrows) of the CZB zinc-binding Cys (pink receptor), leading to weakened ligation and release Zn^2+^, promoting the apo form and chemoattraction. Together, this Zn^2+^ sensory mechanism serves to enable bacteria to navigate to permissive redox environments suitable for their metabolism. CZB-regulated diguanylate cyclases could mediate a similar fitness advantage through increasing adhesion, aggregation, and biofilm formation in response to elevated redox potential.

Based on these data, we propose a new mechanistic model in which McpA senses Zn^2+^ as a secondary messenger of environmental redox state (Figure 6). Oxidizing conditions driven by O_2_ or ROS shift zinc speciation toward insoluble, biologically unavailable zinc precipitates, thereby depleting the soluble Zn^2+^ pool sensed by McpA, promoting an *apo* receptor state and chemoattraction, ultimately serving to drive attraction of the bacteria toward environments with appropriate redox potential permissive for survival (Figure 6). As the structural elements required for this sensing mechanism are conserved across all CZB orthologs, and because the ability to identify favorable redox environments is presumably advantageous across diverse ecological niches, we propose that redox taxis is the broadly conserved and ancestral function of CZB-domain signaling proteins.

## Discussion

At an early period of evolution, bacteria were confronted with a dramatic elevation in oxygenation, with both enriched O_2_ and ROS levels imposing new selective pressures through their propensity to induce oxidative damage (1–3, 6). CZB proteins provide an interesting window into how primordial bacteria began to sense the local redox landscape and seek oxidants for their metabolic benefit (7, 8). Because CZB proteins are estimated to have originated before the GOE, the subsequent rise in atmospheric O_2_ may have conferred a selective advantage on this mode of redox sensing, contributing to the widespread conservation of CZB proteins across bacterial phyla (11, 17).

Cells exert tight control over cytosolic redox reactions and survive within a relatively narrow regime of tolerated redox stress, which differs among cell types and species that inhabit different environments (6–8, 10). Thus, it is conceivably of general importance for bacteria to possess means to measure local redox potential and identify niches suitable for their particular metabolism. In terms of oxidants mediating metabolic advantage, an especially pertinent example is O_2_, but there is an emerging understanding that at low levels certain ROS can also serve as electron donors and promote metabolism; others may serve as cues that do not themselves impart growth advantages but signal favorable environments, such as damaged tissue, or generate useful metabolites such as nitrates or tetrathionate (12, 20, 23, 25, 48). As opposed to responding only to specific ROS, CZBs offer bacteria a generalized mechanism of redox sensing through exploiting the high sensitivity of Zn^2+^ solubility, using the concentration of soluble, bioavailable Zn^2+^ as a proxy for environmental redox potential (11, 12, 61, 62). The mechanism proposed here has no clear parallels across biology, as far as we are aware, potentially reflecting the unique Cys-His-His-His tetrahedral coordination motif found only within this family of proteins.

A prevalent mechanism by which Zn^2+^ binding is coupled to redox chemistry is through cysteine ligation responsive to direct oxidation and disulfide formation, as represented by zinc-finger proteins, heat-shock proteins, and RsrA (63–67). These systems are generally components of oxidative stress responses and transcriptional programs that protect against cellular damage (7, 68, 69). Thus, whereas other Zn^2+^-based redox sensors primarily function as oxidative stress detectors that initiate protective responses, CZBs appear uniquely adapted to help cells exploit the metabolic value of oxidants. Indeed, the representative facultative anaerobic system studied here mediates rapid bacterial chemoattraction to O_2_ sources as well as low concentrations of ROS that can confer metabolic advantages.

### A revised model of sensing by CZB protein domains

We propose that CZB domains function as sensors of exogenous redox potential, mediating both aerotaxis and redox taxis through a common Zn^2+^-dependent sensory mechanism in which redox-dependent changes in the bioavailable Zn^2+^ pool serve as a secondary messenger, reporting the redox state of the local environment. Importantly, this mechanism does not rely on Zn^2+^ undergoing oxidation or reduction. Rather, environmental redox chemistry alters the speciation and bioavailability of zinc by shifting the equilibrium between soluble Zn^2+^ and insoluble zinc-containing precipitates, and McpA senses the resulting changes in the soluble Zn^2+^ pool. This model provides a potentially reconciliatory explanation for prior work that either provided conflicting data or results disconnected from a clear biological role. Many of these discrepancies may boil down to methodology and testing of ROS at levels higher than is physiologically relevant, and we have added a few new dimensions to understanding this behavior.

By conducting titrations of HOCl across several orders of magnitude, we in fact find a bimodal response in that extremely high source concentrations do promote chemorepulsion but lower concentrations within more physiologically relevant regimes promote chemoattraction across HOCl, H_2_O_2_, and ROOH (Figure 1) (31). These findings clarify the concentration dependence of the response, demonstrating that chemoattraction occurs only at ROS concentrations below those that cause bactericidal effects, thereby appearing to resolve the paradox of bacterial attraction to lethal oxidants. Further, these nanomolar-to-micromolar ROS concentrations elicit clear changes in swimming behavior consistent with chemoattraction responses in the form of reduced tumbling rates and increased migration (Figure 2, Figure S2).

While swimming assays in homogenous effector baths have frequently been used as proxies for chemotaxis responses, particularly for bacteria in which other types of chemotaxis assays are technically challenging, we were conscious of the limitations in interpreting these behaviors without orthogonal experiments that directly visualize bacterial localization to a point source of the effector. In assessing the function of McpA in redox taxis, the swimming data paired with CIRA shows clearly how the distribution of the bacterial population changes over time in response to the treatment source. Further, by employing automated tracking, the swimming analyses are based upon assessment of thousands of tracks pooled from different experiments such that we can have greater confidence in our measurements of how ROS, Zn^2+^ levels, and O_2_ impact swimming, and how this relates to bacterial localization. Our new mechanistic findings also provide an explain for our previous observation that depletion of host-derived HOCl had no effect on McpA-mediated fitness during colitis, as McpA responds not only to HOCl but also to hydroperoxides and elevated O_2_, such that elimination of a single attractant signal would be insufficient to abolish McpA signaling (22, 70).

Since McpA senses exogenous redox potential through changes in the soluble, bioavailable Zn^2+^ pool, and also the zinc-binding Cys thiolate can be directly oxidized by high levels of HOCl, for such host-associated pathogens that elicit strong inflammatory responses like *Salmonella* that encounter high concentrations of neutrophil-derived HOCl, both mechanisms could be at play (Fig. 6) (23, 24, 29, 30). Based on earlier work we would expect direct cysteine oxidation to approximate the behavior of the C272A mutant, where the cysteine disengages the Zn^2+^ ligand, lowering the affinity of the zinc-binding site and promoting release (11, 12, 14). This may act as an additional regulatory mechanism for sensory tuning for the subset of CZB-possessing pathogens for which host-derived HOCl might serve as an important cue during pathogenesis.

More broadly, and likely of greatest relevance to the diverse bacteria that possess CZB proteins, the defining chemoselectivity of CZBs may be their ability to distinguish soluble Zn^2+^ from insoluble zinc species. Using simple Nernst-based estimates as a heuristic framework, we compared the theoretical magnitude of effective redox perturbations detected by the CZB Zn^2+^-dependent mechanism with those of more canonical cysteine-, flavin-, and heme-based redox sensors. These estimates suggest that McpA is highly sensitive to changes in environmental redox potential: a change in HOCl from 0–5 nM corresponds to an apparent ΔE_h_ of ~80 mV (71), while a shift from 1% to 21% O_2_ corresponds to ~20 mV for the O_2_/H_2_O couple (72). These values fall within the same general order of magnitude as reported operating ranges for several established redox-responsive systems, including ArcB (~20–50 mV) (73), OxyR (~30 mV) (74), and Yap1/Orp1 (~30 mV) (75), suggesting that CZB-mediated responses accomplish redox sensitivity on par with that of other systems through a wholly different sensory mechanism.

A potential advantage of the CZB mechanism is that it integrates multiple environmental variables that influence respiration, including oxygen availability and ROS, into a single intracellular readout through changes in labile Zn^2+^. Rather than sensing individual oxidants, CZBs instead appear to monitor the cumulative effects of environmental redox chemistry on zinc speciation and the size of the soluble, bioavailable Zn^2+^ pool. More broadly, this signaling principle may extend beyond CZB domains to other zinc-coordinating redox-responsive proteins, such as zinc-finger domains. Whereas oxidative regulation of these systems has traditionally been attributed to direct cysteine oxidation, disulfide formation, and Zn^2+^ release (63, 65, 67, 68), our findings suggest that redox-dependent changes in soluble Zn^2+^ availability can regulate metallosensory proteins by shifting the equilibrium between their Zn^2+^-bound and Zn^2+^-free states, identifying a potentially general mechanism by which environmental redox chemistry is coupled to cellular signaling.

## Materials and Methods

### Preparation of motile Salmonella Typhimurium

Bacterial strains and plasmids used in this study are listed in Table S1. To prepare cells for motility and chemotaxis assays, bacteria were cultured overnight in 2 ml of tryptone broth (TB) and, for antibiotic selection, 50 μg/ml ampicillin (TB + Amp), at 30 °C. 50 μl of overnight culture was used to inoculate 25 ml of fresh TB + Amp the next day, followed by shaking for 3 hours to reach approximately A_600_ of 0.5. Bacterial cultures were centrifuged at 1,500 g for 20 minutes and resuspended into chemotaxis buffer (CB) containing 10 mM potassium phosphate, 10 mM sodium lactate, at pH 7. Cultures were then diluted to approximately A_600_ = 0.2 and rocked for 30–60 minutes before experimentation. For hypoxic CIRA experiments, the same protocol was used except for culturation was performed in an AnaeroJar 2.5L Jar (Oxoid, Basingstoke, UK) and a ThermoFisher CampyGen microaerophilic 2.5L Sachet (Waltham, Massachusetts, USA) with shaking at 37 °C.

### In vitro growth with ROS challenge

Wild-type *Salmonella* Typhimurium IR715 was incubated overnight in 25 ml of Luria-Bertani (LB) + Amp. The following day, cultures were pelleted by centrifugation at 2000 g for 20 minutes and resuspended in minimal media (MM) containing 47 mM Na_2_HPO_4_, 22 mM KH_2_PO_4_, 8 mM NaCl, 2mM MgSO_4_, 0.4% glucose (w/v) 11.35 mM (NH_4_)_2_SO_4_, and 100 μM CaCl_2_. 5 μl of the culture, adjusted to A_600_ = 2 was then used to inoculate fresh solutions of MM and additives of ROS (HOCl, H_2_O_2_, ROOH) in phosphate buffered saline (PBS), for a final volume of 200 μl per well in a 96-well microtiter plate. A Clariostar Plus plate reader (BMG Lab Tech, Ortenberg, DE) was used to monitor cell growth while shaking at 37° via A_600_ readings every 5 minutes for a period of 24 hours. Hypoxic growth experiments were conducted similarly under control of an atmospheric pressure unit (BMG Lab Tech, Ortenberg, DE) maintaining 10% CO_2_ and 1% O_2_ for the duration of data collection.

### Chemosensory injection rig assay (CIRA)

The CIRA apparatus is described previously (76). Briefly, treatment solutions are injected through a Femtotip II glass microcapillary (Eppendorf, Hamburg, DE) controlled with a MP-285 micromanipulator (Sutter, Novato, CA) into a 50 μl pond of motile bacteria on a 10-well slide (MP Biomedicals, Solon, OH). To generate a microgradient of effector, a constant flow of approximately 300 fL/min from the microcapillary was induced by applying compensation pressure (P_c_) of 35 hPa. Treatment solutions were made fresh each day, diluted into CB, and adjusted to pH 7. Bacterial responses were imaged with an inverted Nikon Ti2 Eclipse microscope (Tokyo, JP) with multichannel fluorescence imaging capabilities. In experiments done under hypoxic conditions, the unit was housed within a Coy Lab chamber (Grass Lake, Michigan, USA), controlling temperature and atmospheric to be 1% O_2_, 10% CO_2_ and 37 °C.

### CIRA microgradient modeling

The CIRA microgradient was modeled according to the 3-dimension diffusion equation, as described previously (34). Diffusion coefficients for effectors were 6.5 × 10^−6^ cm^2^/sec for HOCl and H_2_O_2_, and 4.5 × 10^−6^ cm^2^/sec for cumene hydroperoxide, based on prior work (77–79).

### Quantification of CIRA data

Videos of chemotactic responses were quantified as described previously (34). The number of cells in each frame was calculated by determining a fluorescence intensity ratio per cell for frames pretreatment and calculating fluorescence intensity over time using the ‘plot profile’ function of ImageJ. Cell numbers were normalized to a baseline of ‘100%’ at the start of treatment (shown as time 0). Distribution of the bacterial population was quantified through use of the ‘radial profile’ ImageJ plugin. Radial distribution data were normalized by setting the field of view periphery as the baseline of ‘onefold’, which we defined as 280 μm distance from the source. For experiments with non-fluorescent cells, equivalent procedures were performed using phase-contrast data and enumeration of cells over time using a MATLAB-based tracking software, as described previously (42). For hypoxic CIRA data, a bleach correction was applied to all videos due to photobleaching of cells, using the “Bleach Correction” plugin in ImageJ (80).

### Bacterial swimming assay

Bacteria were cultured as described above and back-diluted to A_600_ = 0.025 in a solution of CB or effector diluted into CB. This density allows for high quality tracks of individual bacteria to be obtained with minimal overlapping of swimming trajectories. For each experiment, a fresh pond of 50 μl of motile bacteria was mounted on a 10-well slide (MP Biomedicals, Solon, OH). Bacteria were imaged over a 30 s period. Bacteria were then tracked using TrackingGUI (42), with bpfiltsize set to 20, the threshold option set to 2 (STD of thresholds), and the default threshold set at 4.0. Tracks were culled on the basis of the STD of position = 2, and the track length = the frame rate of the video (typically 25 fps), thereby selecting only motile cells and those that were monitored for at least one full second. Linear migration for the first second of each track was determined by:

$$\text{Migration}\;=\sqrt{{\left(d_{x}\right)}^{2}+{\left(d_{y}\right)}^{2}}$$

Where d_x_ and d_s_ are the differences in x and y pixel coordinates between the first and last valid frame within the first one-second window of each track. Average speed for the first second of each track was calculated as:

$$\text{Average}\;\text{Speed}\;=\frac{\text{total}\;\text{path}\;\text{length}}{\text{elapsed}\;\text{time}}$$

Where the total path length is every frame-to-frame Euclidean step distance within the first second of each track, and elapsed time is the actual elapsed time of those frames. Finally, tumble detection was performed based on the approach described in Johnson et al. in 2024 (81). Briefly, instantaneous translational speed and angular speed were calculated from frame-to-frame positional differences and smoothed using a Savitzky-Golay filter of polynomial order 2 with a window automatically scaled to approximate 200 ms. Tumble candidates were identified as local minima in the smoothed speed trace. Each candidate was retained only if the difference between the adjacent peak speed and the minimum speed was at least 70% of the minimum speed itself. Candidates were then subject to two additional confirmation criteria. First, angular speed at the candidate frame (±1 frame) was required to exceed the track mean angular speed by at least 20%, consistent with the direction change expected during a genuine tumble event (81). Second, the speed at the minimum had to exceed 3.0 μm/s, to exclude artifactual tumbles arising from bacteria that stopped due to contact with a surface or exit from the focal plane. Tumble frames were delineated as those falling between the flanking local maxima whose smoothed speed remained within 30% of the speed range between the minimum and the surrounding peaks. Tumble frequency was then calculated for each bacterial swimming track as the total number of confirmed tumbles divided by total track duration in seconds.

### Free Zn^2+^ Assay

1 mM solutions of ZnSO_4_ were prepared under both hypoxic and normoxic conditions +/− the addition of ROS. After 3 hours of incubation, insoluble zinc species precipitated within the tube and were removed by centrifugation. The supernatant was subsequently removed and assayed with Zinpyr-1 (Santa Cruz Biotechnology, Dallas, TX, USA), a fluorescent probe that specifically binds Zn^2+^ (82, 83). From there, the fluorescence of the solutions from 500–600 nm was determined based on a standard curve to determine the concentration of Zn^2+^ in each solution.

### Statistical Analysis

Data from replicate experiments were averaged and interpreted on the basis of their mean and standard error of the mean. Where relevant, p-values were calculated using Kruskal-Wallis test followed by Dunn’s multiple comparison tests or by unpaired t-tests, with significance determined at p<0.05.

## Supplementary Material

Supplement 1

## Significance Statement

We report a previously unknown mechanism of redox sensing that operates through changes in the bioavailability and speciation of Zn^2+^, enabling bacteria to detect changes in exogenous redox potential with high sensitivity and navigate redox gradients. The broad phylogenetic distribution of chemosensory zinc-binding (CZB) domains suggests that this zinc-dependent sensory mechanism is an ancient and widespread strategy for detecting environmental redox gradients that arose early in bacterial evolution and may have been subsequently shaped or expanded in response to increasing atmospheric oxygen associated with the Great Oxygenation Event.
