# Supplementary material for: Zn2+ as a secondary messenger for exogenous redox potential sensed through Chemosensory Zinc-Binding (CZB) protein domains
Source: bioRxiv. 2026 Jul 7:2026.07.03.735341. Preprint. [Version 1] doi: 10.64898/2026.07.03.735341 (PMC13370339; doi:10.64898/2026.07.03.735341)
Supplement: Supplement 1 [file NIHPP2026.07.03.735341v1-supplement-1.pdf]

bioRxiv preprint doi: https://doi.org/10.64898/2026.07.03.735341; this version posted July 7, 2026. The copyright holder for this preprint (which was not certified by peer review) is the author/funder, who has granted bioRxiv a license to display the preprint in perpetuity. It is made available under a CC-BY 4.0 International license.

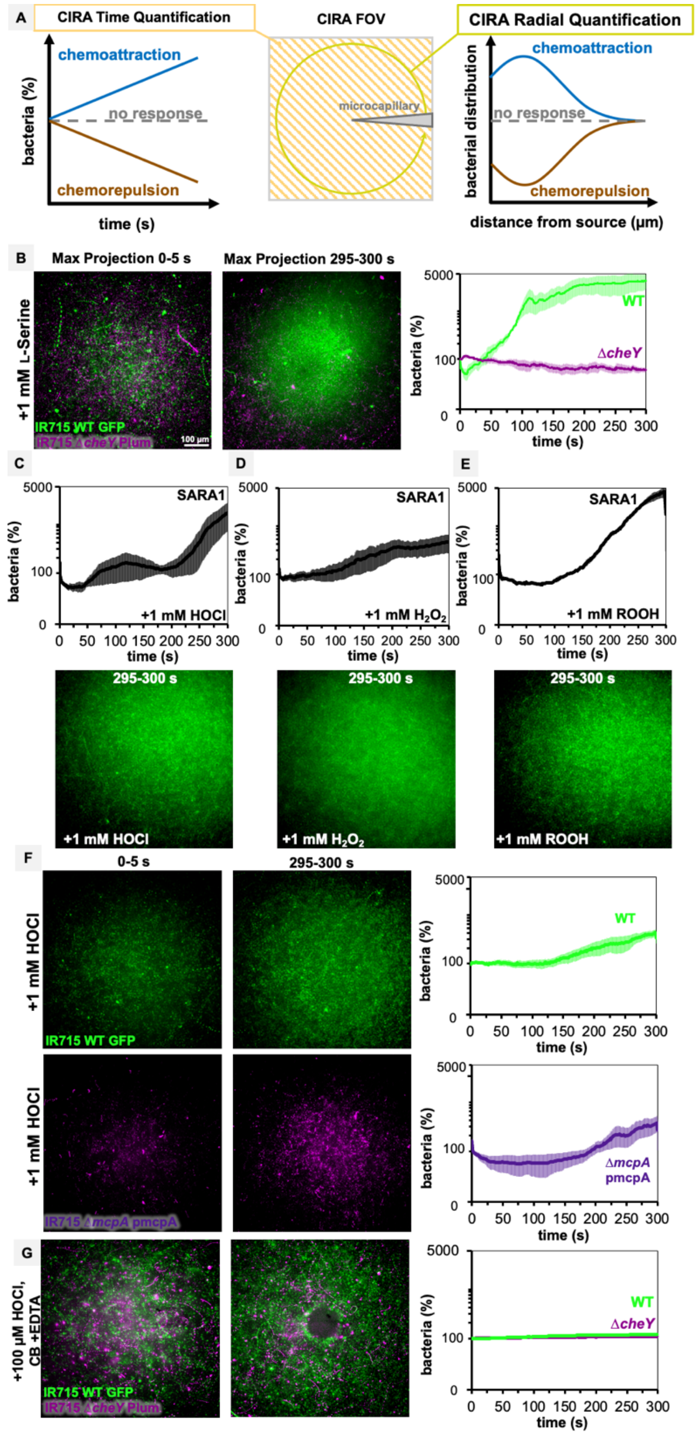

Fig. S1. CIRA quantification and experimental controls. A. Two methods of quantifying data from CIRA. B. Dual-channel imaging of chemotactic responses to 1 mM L-Serine by WT *S.* Typhimurium IR715 (green) and a Δ*cheY* mutant (purple). Shown are max projections at times 0-5 s and 295-300 s post-treatment, and enumeration of bacteria within the field of view. C-E. Responses of the human clinical *S.* Typhimurium isolate SARA1 to 1 mM ROS injections, as after 5 minutes. F. Dual-channel imaging of chemotactic responses to 1 mM HOCl by WT (green) versus a Δ*mcpA* pmcpA complementation mutant (red); bacteria were co-cultured in the same experiment but channels are separated for clarity. G. Dual-channel imaging of chemotactic responses to 1 mM HOCl by WT (green) and a Δ*cheY* mutant (purple) cultured in chemotaxis buffer with 100 µM EDTA added, as indicated. All data are means and error bars are standard error of the mean (SEM, n=3-5).

bioRxiv preprint doi: https://doi.org/10.64898/2026.07.03.735341; this version posted July 7, 2026. The copyright holder for this preprint (which was not certified by peer review) is the author/funder, who has granted bioRxiv a license to display the preprint in perpetuity. It is made available under a CC-BY 4.0 International license.

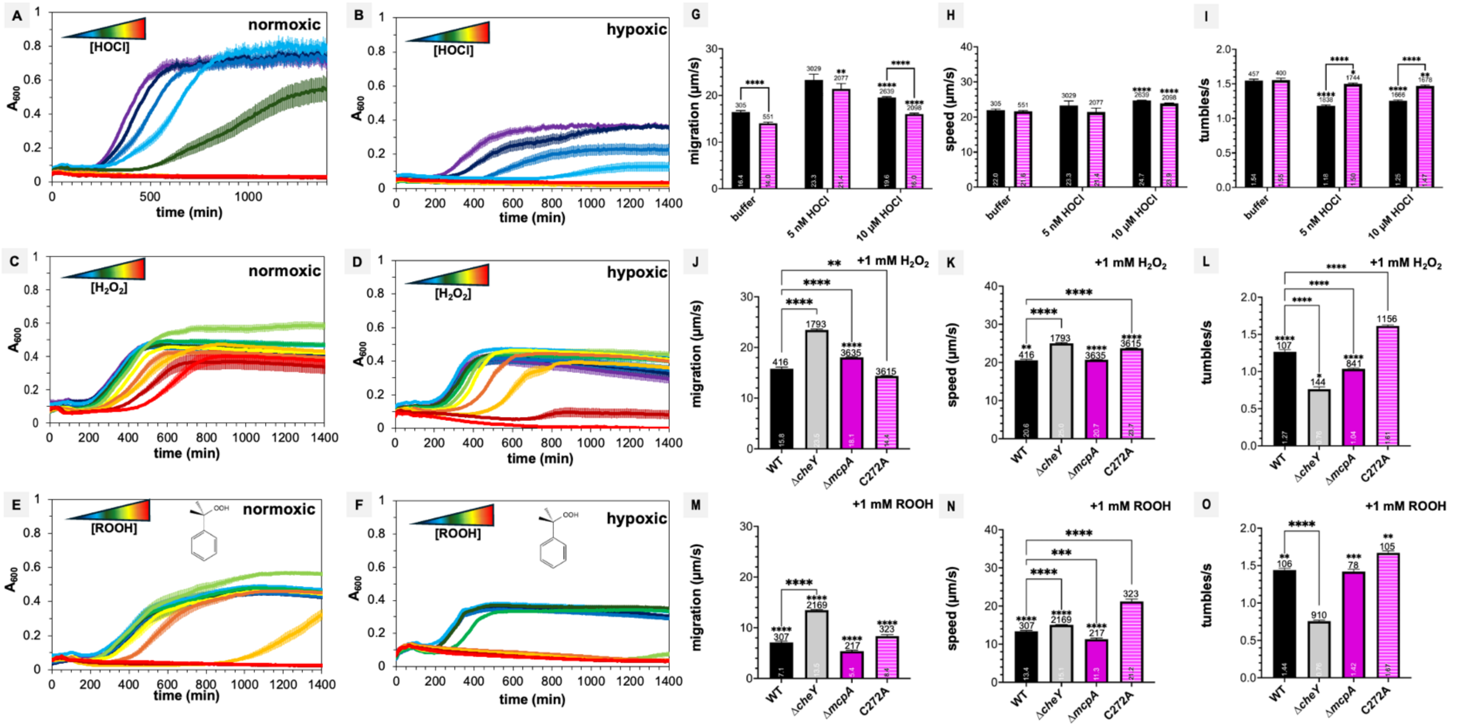

Fig. S2. Impacts of neutrophil ROS on bacterial growth and swimming. A-F. Growth of *S.* Typhimurium IR715 with increasing concentrations of HOCl (A-B), H_2O_2 (C-D), and ROOH (E-F) under normoxic and hypoxic conditions, as indicated (n=24). C-E. Quantification of bacterial migration and speed over the first second of each track as well as tumbles per second based on bathing *Salmonella* WT and C272A in various HOCl concentrations dissolved in CB. J-O. Quantification of bacterial migration and speed over the first second of each track as well as tumbles per second based on bathing *Salmonella* WT and mutant cells in 1 mM H_2O_2 (J-L) or 1 mM ROOH (M-O) dissolved in CB. All data are means and error bars are standard error of the mean (SEM). Quantification of bacterial swimming. Statistical significances between strains, denoted by bars within each treatment group, were calculated using a Kruskal-Wallis test followed by Dunn's multiple comparison tests, compared to the WT strain within that treatment group. Statistical significances for strain differences compared to buffer treatment version of the same strain are denoted by stars above the number of tracks and were calculated by unpaired t-tests (not significant, not noted; * $p < 0.05$, ** $p < 0.01$, *** $p < 0.001$, **** $p < 0.0001$).

bioRxiv preprint doi: https://doi.org/10.64898/2026.07.03.735341; this version posted July 7, 2026. The copyright holder for this preprint (which was not certified by peer review) is the author/funder, who has granted bioRxiv a license to display the preprint in perpetuity. It is made available under a CC-BY 4.0 International license.

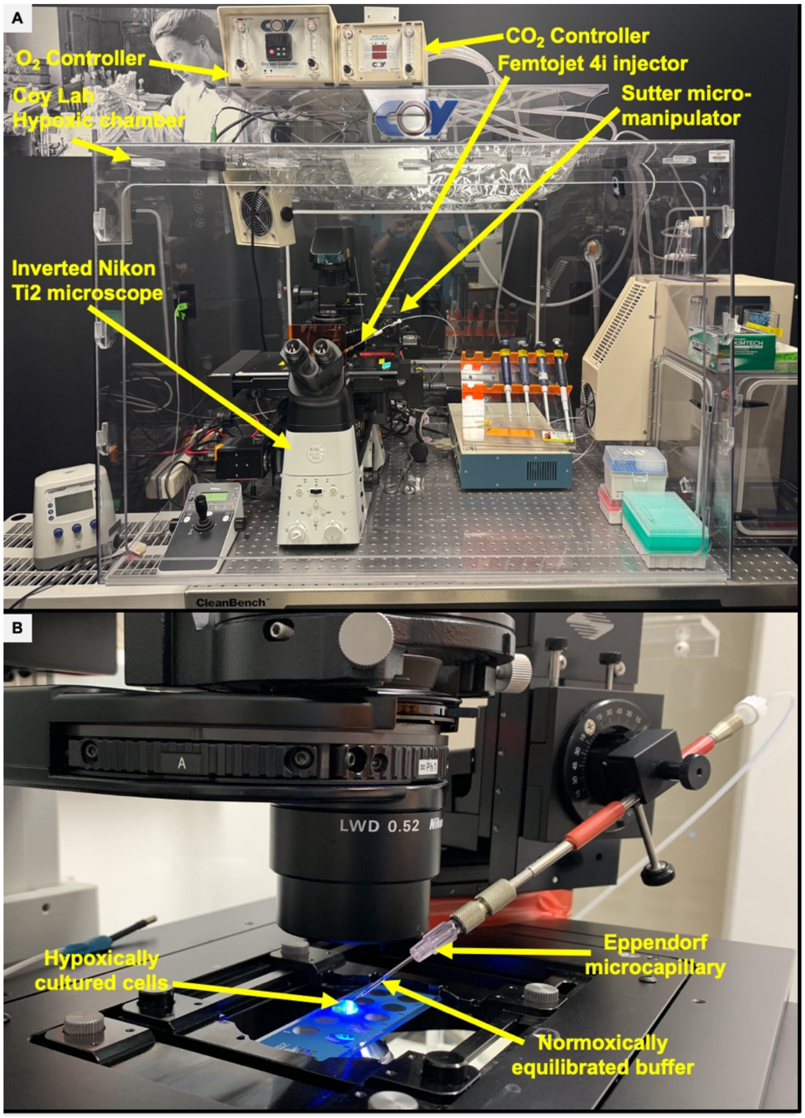

Fig. S3. Custom hypoxic CIRA setup for aerotaxis live-imaging. A. Overview of the custom hypoxic imaging system. **B.** Close-up of the microscope stage during a CIRA experiment, showing hypoxically cultured cells on the imaging surface with an Eppendorf microcapillary positioned to inject normoxically equilibrated buffer. This integrated system enables real-time visualization of aerotactic responses while cells are maintained under enteric-relevant oxygen conditions (1% O_2, 10% CO_2).

bioRxiv preprint doi: https://doi.org/10.64898/2026.07.03.735341; this version posted July 7, 2026. The copyright holder for this preprint (which was not certified by peer review) is the author/funder, who has granted bioRxiv a license to display the preprint in perpetuity. It is made available under a CC-BY 4.0 International license.

Supplementary Videos

Supplementary Video 1: Responses of WT *S.* Typhimurium IR715 and Δ*cheY* to 1 mM ROS at 10X speed. Experiments were conducted under normoxic conditions at 22°C at 10X speed. Full video length is 5 minutes. Viewable at: https://youtu.be/t56cjShQDZM

Supplementary Video 2: Responses of WT *S.* Typhimurium IR715 and Δ*mcpA* to 1 mM ROS at 10X speed. Experiments were conducted under normoxic conditions at 22°C. Full video length is 5 minutes. Viewable at: https://youtu.be/iOXzixrMu7M

Supplementary Video 3: Responses of WT *S.* Typhimurium IR715 and C272A to 1 mM ROS at 10X speed. Experiments were conducted under normoxic conditions at 22°C. Full video length is 5 minutes. Viewable at: https://youtu.be/ElE2J3BfM2s

Supplementary Video 4: Responses of WT *S.* Typhimurium and C272A to zinc-altered buffers (Chelex = zinc-chelated, Chelex +1 mM $ZnSO_4$ = zinc-chelated with added Zn^{2+}, +1 mM ZnO = zinc-chelated with added oxidized zinc) at 10X speed. Experiments were conducted under normoxic conditions at 22°C. Full video length is 5 minutes. Viewable at: https://youtu.be/gfEmb01Rg2s

Supplementary Video 5: Responses of WT *S.* Typhimurium IR715, Δ*mcpA*, and C272A point mutant to injected normoxically equilibrated (+O_2) chemotaxis buffer at 10X speed. Bacteria were cultured under hypoxic (1% O_2) conditions at 37 °C. Experiments were conducted under hypoxic (1% O_2 + 10% CO_2) conditions at 37°C. Full video length is 5 minutes. Viewable at: https://youtu.be/fbALQ6STMWA

Supplementary Video 6: Responses of WT *S.* Typhimurium IR715, Δ*mcpA*, and C272A point mutant to injected hypoxically equilibrated (-O_2) chemotaxis buffer at 10X speed. Bacteria were cultured under hypoxic (1% O_2) conditions at 37 °C. Experiments were conducted under hypoxic (1% O_2 + 10% CO_2) conditions at 37°C. Full video length is 5 minutes. Viewable at: https://youtu.be/3jByzjDtuEs

bioRxiv preprint doi: https://doi.org/10.64898/2026.07.03.735341; this version posted July 7, 2026. The copyright holder for this preprint (which was not certified by peer review) is the author/funder, who has granted bioRxiv a license to display the preprint in perpetuity. It is made available under a CC-BY 4.0 International license.

Table S1. Bacterial strains, plasmids and chemicals used in this study.

Strains	Description	Source
***S. enterica* Typhimurium**		
IR715	Nalidixic acid-derivative of ATCC 14028	(84)
FR4	IR715 *tsr*::pFR3 (Cm^R)	(84)
FR5	IR715 *aer*::pFR2 ($Carb^R$)	(84)
FR13	IR715 *cheY*::Tn*10* (Tet^R)	(84)
FR35	IR715 *mcpC*::Cm^R	(84)
FR36	IR715 *mcpB*::Kan^R	(84)
FR37	IR715 *mcpA*::Kan^R	(84)
FR42	IR715 *trg*::Cm^R	(84)
C272A	IR715 *mcpA*::Kan^R/glms::PmcpA-mcpA C272A TT Cm	(22)
pmcpA	IR715 *mcpA*::Kan^R/glms::PmcpA-mcpA TT Cm	(22)
***S. enterica* SARA1**		(85)
**Plasmids**		
pXS-sfGFP	pGEN-mcs with a modular sfGFP expression scaffold (Amp^R)	(34, 35)
pXS-mPlum	pGEN-mcs with a modular mPlum expression scaffold (Amp^R)	(34, 35)
**Chemicals**		
cumene hydroperoxide, 80%	Cat. No.: 349962500	Thermo Scientific (Waltham, MA)
hydrogen peroxide, 30%	Cat. No.: 2186-03	Avantor (Radnor Township, PA)
sodium hypochlorite, 11-15% available chlorine	Cat. No.: 03369.A9	Thermo Scientific (Waltham, MA)
Chelex-100 Resin	Cat. No.: 142-1253	Bio-Rad Laboratories (Hercules, CA)
Zinpyr-1	Cat. No.: sc-213162	Santa Cruz Biotechnology (Dallas, TX)
